# d-Amino Acid Pseudopeptides as Potential Amyloid-Beta Aggregation Inhibitors

**DOI:** 10.3390/molecules23092387

**Published:** 2018-09-18

**Authors:** Banafsheh Mehrazma, Stanley Opare, Anahit Petoyan, Arvi Rauk

**Affiliations:** Department of Chemistry, University of Calgary; Calgary, AB T2N 1N4, Canada; bmehrazm@ucalgary.ca (B.M.); skaopare@ucalgary.ca (S.O.); anahit.petoyan@gmail.com (A.P.)

**Keywords:** Alzheimer’s, amyloid-beta, inhibitors, d-amino acids, molecular dynamics, umbrella sampling

## Abstract

A causative factor for neurotoxicity associated with Alzheimer’s disease is the aggregation of the amyloid-β (Aβ) peptide into soluble oligomers. Two all d-amino acid pseudo-peptides, SGB1 and SGD1, were designed to stop the aggregation. Molecular dynamics (MD) simulations have been carried out to study the interaction of the pseudo-peptides with both Aβ_13–23_ (the core recognition site of Aβ) and full-length Aβ_1–42_. Umbrella sampling MD calculations have been used to estimate the free energy of binding, ∆G, of these peptides to Aβ_13–23_. The highest ∆G_binding_ is found for SGB1. Each of the pseudo-peptides was also docked to Aβ_1–42_ and subjected up to seven microseconds of all atom molecular dynamics simulations. The resulting structures lend insight into how the dynamics of Aβ_1–42_ are altered by complexation with the pseudo-peptides and confirmed that SGB1 may be a better candidate for developing into a drug to prevent Alzheimer’s disease.

## 1. Introduction

Alzheimer’s disease (AD) is the most common form of dementia, affecting more than 35 million people worldwide [1]. Although it has been over 100 years since the discovery of the disease, there has been no effective drug that can slow or stop the progress of AD [2,3].

One of the extensively studied pathways of neurodegeneration of AD is based on the β-amyloid peptide (Aβ) hypothesis [1,4,5]. Soluble monomeric Aβ peptides misfold into well-ordered hydrogen-bonded β-sheet-rich protein aggregates [6,7,8]. The produced soluble oligomers eventually deposit as amyloid plaques. A high level of Aβ peptide with 38–43 residues exists in AD brains [9].

Aβ oligomers are more cytotoxic than their monomeric or fibrillar species [1,9,10,11,12,13,14,15]. Conversion of the amyloid structures to less active species will lead to less toxic forms of the protein [14,15]. This implies that any strategy which leads to a lesser number of toxic oligomers would be beneficial.

Inhibition of amyloid aggregate formation is the main goal of many research groups seeking a treatment for AD [10]. The oligomers of Aβ contain high amounts of β-sheets [16,17]; the antiparallel β-sheet motif has been identified in oligomers of Aβ_42_ by ATR (attenuated total reflection)-FTIR (Fourier transform infrared) spectroscopy [16].

One of the prominent sites of self-binding in Aβ is in the sequence, Aβ_16–20_ (KLVFF), proceeding via anti-parallel β-sheet formation [6,8,18,19]. Based on ITC (isothermal calorimetry) results for fibrilization of the sequence, Aβ_16–24_ (KLVFFAEDV) with both termini amidated, the free energy (∆G) of fibril formation was determined to be ∆G = −37.5 kJ/mol [6].

In addition to Aβ_16–20_, other residues have been reported to have crucial roles in aggregation. For example, the charged group residues are thought to add to the binding propensity of the peptide by making salt-bridges. Relevant to this matter, Glu22 and Asp23 are known to be two of the key residues to make salt-bridges which further promote the aggregation of Aβ [20,21,22,23]. The nearby histidine residues His^13^His^14^ are well-known to bind to metals such as copper and cause related neurotoxicity in the AD brains through reactive oxygen species (ROS) generation [24,25,26,27,28]. Having an interest both in metal involvement and aggregation through β-sheet formation, we consider the sequence, Aβ_13–23_ (HHGKLVFFAED), designated as R, as a suitable small model of full length Aβ. In this paper, the focus is on the aggregation, or more specifically, inhibition of aggregation of Aβ with two model pseudo-peptides.

Previously, we have studied four classes of pseudo-peptides that bind to R with high affinity: SGA, SGB, SGC, SGD [23,29,30,31]. The sequences of the selected pseudo-peptides in each group is as following:

SGA3 = *N*-Acetyl-Daba1-Orn2-MeLeu3-Phe4-MePhe5-Leu6-Ala7-Glu8-NH_2_

SGB1 = *N*-Acetyl-daba1-orn2-leu3-mephe4-phe5-mephe6-leu7-glu8-NH_2_

SGC1 = *N*-Acetyl-Glu1-Ala2-MePhe3-Phe4-MePhe5-Leu6-Orn7-Daba8-NH_2_

SGD1 = *N*-Acetyl-glu1-leu2-mephe3-phe4-mephe5-leu6-orn7-daba8-NH_2_

where the lower case designates the d-amino acids and Daba = diaminobutyric acid, Orn = ornithine, MeLeu = *N*-methylleucine, MePhe = *N*-methylphenylalanine. All these pseudo-peptides mimic the R section of the Aβ peptide; having complimentary charged residues between the hydrophobic core. The all l-amino acid SGA and SGC bind to R in antiparallel and parallel mode, respectively. The all-d-amino acid SGB and SGD bind to R in an antiparallel and parallel mode, respectively. The characteristic of all of these pseudo-peptides is the use of the unnatural amino acids. The use of d-amino acids and unnatural amino acids in the peptides aids the evasion from the immune system and enzymatic degradation and should increase the cellular half-life of the pseudo-peptides [32,33,34,35,36]. The *N*-methylated groups; MeLeu and MePhe were used to stop β-sheet propagation [37,38,39,40,41]. The methyl groups will interfere with the β-sheet interaction by hindering the hydrogen bonding in the backbone. The all-d-amino acids, SGB1 and SGD1 have not been investigated in detail. In this article, we will focus on the interaction of the all-d-amino acid pseudo-peptides; SGB1 and SGD1, with a central region of Aβ, R = Aβ_13–23_ and with full-length Aβ_1–42_.

The use of the d-amino acid peptides is not unique to the present study [42]. In 1996, Soto et al. discovered that the 11-mer d-amino acid peptide, d-iAβ1 has similar β-sheet breaker properties as its l-amino acid version L-iAβ1 with sequence of RDLPFFPVPID [32]. Similarly, in 1999, Findeis et al. found that the all-d-amino acyl analogue peptide acid (PPI-433) and amide (PPI-457) of a lead compound for Aβ anti-aggregation, have similar activity as that of the all l-amino acyl analogue (cholyl-LVFFA-OH).^34^ Findeis et al. had reported that cholyl-modified compounds are being eliminated as if they were endogenous bile components [34]. A good incentive to promote the research on d-peptides comes from the work of Chalifour et al. in 2003, in which they realized the all d-amino acid sequence klvffa has a higher propensity to inhibit the fibril formation of Aβ than its l-enantiomeric form, with increased cell viability in SH-SY5Y cells [43]. Another all-d-amino acid peptide with the sequence of qshyrhispaqy binds specifically to Aβ with K_D_ of 0.4 μM [44]. Although this amino acid reduces the size of the Aβ aggregates, the Willbold group claimed reduced cytotoxicity of the peptide in vitro [45]. Both in vivo and in vitro studies, performed by the Willbold group, verified that an orally bioavailable d-amino acid peptide designated as D3 (rprtrlhthrnr) interferes with the aggregates of Aβ and reduces the Aβ cytotoxicity [46,47]. They had hypothesized that D3 renders the aggregates to non-amyloidogenic and nontoxic species [47]. A retro-inverso peptide RI-OR2, with the sequence of rGffvlkGr was identified to interact with Aβ with K_D_ of 9–12 μM [48]. Higher anti-Aβ aggregation efficacy with increased cell viability was reported for RI-OR2 compared to the all-l-amino acid OR2 peptide [48].

**Figure 1:**
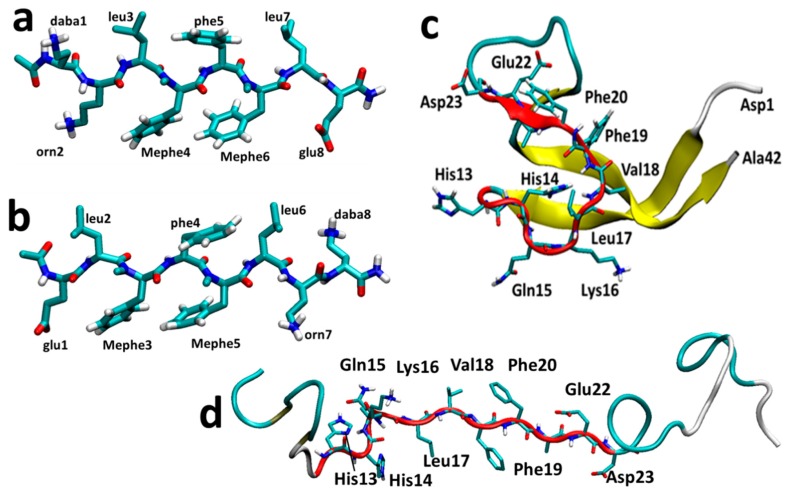
The most stable structures of SGB1 (**a**) and SGD1 (**b**) obtained from the MD simulation. The numbering is from the N-terminal amino-acid. (**c**) Reference geometry of Aβ_42_. (**d**) Extended geometry of Aβ_42_ for docking. The R region of Aβ is highlighted as a red cartoon with side chains.

Here, we will investigate the interaction of SGB1 and SGD1 with R (Figure 1a,b) with use of molecular dynamics simulations. Energy analysis through umbrella sampling calculations is carried out to assess the strength of their binding to R. We also investigate the interaction of the two pseudo-peptides with Aβ_42_, while they interact in the R region of Aβ.

## 2. Methods

VMD software was used for visualization [49]. All molecular dynamics simulations, Steered MD and analysis were performed by GROMACS 4.0.7 or 4.6.5 software [50] and the GROMOS96 53a5 force field [51] (a united aliphatic C-H atom force field). Particle mesh Ewald summation was considered for long range electrostatic interactions. The Fourier spacing was 0.12 nm. For neighbor searching the twin-range approach was chosen. The Van der Waals cut-off and electrostatic cut-off were both 1 nm. The temperature was set to be at 310 K by use of the Nose-Hoover temperature coupling [52,53]. In addition, the pressure was maintained at 1 bar with the use of the Parrinello-Rahman pressure coupling [54,55] with a coupling constant of 1 ps. All bonds were constrained by LINCS algorithm [56].

### 2.1. The Interaction of SGB1 and SGD1 with R (Aβ_13–23_)

The structures and energy data for R monomer and dimers were taken from our earlier work [30,57]. The R monomer model of Aβ monomer is acetylated at the N-terminus, His13 and amidated at Asp23. The dimers of R-SGB1, R-SGD1, SGD1-SGD1 and SGB1-SGB1 were docked with Hex 6.3 [58,59,60,61].

#### 2.1.1. Molecular Dynamics Simulation

Each compound was put in a cubic box with the dimensions of 6 × 6 × 6 nm^3^ and solvated by the simple point charge water model (SPC) [62] R-SGB1 and R-SGD1 have a total charge of zero but for the homodimers, SGB1-SGB1 and SGD1-SGD1, 2 Cl^−^ ions were added to neutralize the system. Next, a 10,000 step steepest descent energy minimization was performed, followed by a 100 ps position-restrained MD simulation. Then an MD simulation of typically 200 ns duration was carried out with a time step of 2 fs, yielding a more or less equilibrated system. To assess the equilibration, RMSD calculation and cluster analysis were performed. For the cluster analysis, an RMSD cut-off of 0.25 nm was used for grouping similar structures (conformations).

#### 2.1.2. Steered MD (MD-SMD) Calculations and Umbrella Sampling Calculation (MD-US)

Steered MD was used to define a dissociation pathway for dimeric complexes. The dimer cluster with the highest population from the MD equilibration was chosen and the monomers pulled apart along the vector defined by their centers of mass (COMs) at a rate of 0.000015 nm/ps for a total of 200 ns, resulting in a further 3.0 nm separation of the two monomers. The output from SMD calculations was then subjected to umbrella sampling calculations. Thirty points (windows), at a separation of 0.1 nm, were selected on the dissociation pathway. At each point, the system was constrained to the reaction path by a harmonic potential with force constant, 1000 kJ/(mol·nm^2^) and equilibrated for 50 ns each. Normally, these parameters allowed a sufficient overlap of the distributions (histograms) to permit WHAM (weighted histogram analysis method) analysis [63]. If needed the additional windows were added in between to ensure smooth overlap between adjacent histograms. The first 10 ns were allowed for further equilibration and the last 40 ns used for the WHAM analysis. The difference between the high and low points of the resulting PMF curve was taken as an estimate of the free energy of binding ∆G_binding_. Error estimates were calculated by use of Bayesian bootstrap analysis [64], with autocorrelation turned on and 100 bootstraps for each window. The error bars shown on the PMF curves are for 1 standard deviation.

For each window, the average number of intermolecular hydrogen bonds (H-bond) between the positively charged residues on one monomer and negatively charged residues on the other monomer were calculated. The H-bond count includes contributions from salt bridges. The averages of the minimum salt bridge distances are calculated separately. When the separation of polar residues is less than 1 nm, we consider a salt bridge to exist. This helps to monitor the separation of the two monomers during the simulation.

### 2.2. The Interaction of SGB1 and SGD1 with Aβ_42_

SGB1 and SGD1 (Figure 1a,b) were docked to Aβ_42_ with AutoDock 4.2 [64]. The pseudo-peptides (PP) were allowed to be flexible in the course of docking and the Aβ_42_ peptide as the macromolecule, was held rigid. The initial structure of Aβ_42_ was retrieved from a 700 ns simulation (Figure 1c) [25]. The R region of this structure was converted into a β-strand manually by GaussView 4.1.2 prior to docking (Figure 1d) in order to bias attachment to this region. The few lowest energy docked poses were selected as starting structures for the MD simulations. Henceforth, we refer to Aβ_42_ simply as Aβ and the pseudo-peptides collectively as PP.

#### 2.2.1. Molecular Dynamics Simulation

Each PP-Aβ complex was placed in a cubic box with the dimensions of 9 × 9 × 9 nm^3^. The initial steps of the simulation, namely solvation, charge neutralization and energy minimization, were as described for the R-SGB1 and R-SGD1 complexes above. The temperature and pressure controls were also as described above. For the purpose of energy analyses described below, three energy groups were assigned for each complex. that is, PP, Aβ and solvent. Each simulation was carried out for about 1 μs. Cluster analyses was performed with a RMSD cut-off of 0.35 nm for grouping similar structures based on backbone atoms. Intermolecular salt-bridge analyses and intermolecular hydrogen bond analyses were performed for select structures (clusters).

#### 2.2.2. Relative Energy Determination

Because of the higher flexibility of PP-Aβ complexes, it was not possible to carry out MD-US to evaluate binding energies as it was for PP-R complexes. Instead, we adopt and adapt several approximate schemes based on endpoint sampling to evaluate the free energy change, ∆G_binding_, for the reaction. We note that all terms are ensemble-averaged, where the ensemble corresponds to all frames assigned to a particular conformation by cluster analysis with the criterion RMSD = 0.35 nm:

PP_aq_ + Aβ_aq_ → (PP*_aq_ + Aβ*_aq_) → PP*Aβ*_aq_ ∆G_binding_, (1)

with

∆G_binding_ = G_gas_ (PP*Aβ*) + G_sol_ (PP*Aβ*) − (G_gas_ (PP) + G_sol_ (PP)) − (G_gas_ (Aβ) + G_sol_ (Aβ)) (2)

where the asterisked terms, PP* and Aβ* indicate that the structures are as in the complex, PP*Aβ*_aq_. G_gas_ (PP*Aβ*) contains internal gas-phase electrostatic (i.e., polar) and van der Waals (nonpolar/Lennard-Jones) terms for PP* and Aβ*, V_gas_ (PP*) = V_gas,es_ (PP*) + V_gas,vdW_ (PP*) and V_gas_ (Aβ*) = V_gas,es_ (Aβ*) + V_gas,vdW_ (Aβ*) and the gas-phase interaction between them, V_int_ (PP* − Aβ*) = V_int,es_ (PP* − Aβ*) + V_int,vdW_ (PP* − Aβ*).

Gas-PBSA: In the simplest scheme, Gas-PBSA, all “gas” terms in Equation are calculated from the GROMOS96 53a5 force field and the “sol” terms are calculated by the PBSA procedure using charges from the GROMOS96 53a5 force field. We note that missing from this approximation derived from equation is a detailed accounting of the entropy change, although the ensemble averaging incorporates some aspects of entropy.

LIE-D: In the approximations of Linear Interaction Energy (LIE) theory, it is assumed that a flexible ligand is docked to a rigid protein and that the binding free energy ensues entirely from the difference in surroundings of the bound and unbound ligand; both bound and unbound endpoints are sampled, the latter only for the ligand. All terms of equation are derived from the GROMOS96 53a5 force field. The standard LIE (i.e., LIE-S) equation becomes:

∆G_binding_^LIE-S^ ≈ V_int_ (PP* − Aβ*) + G_sol_ (PP*Aβ*) − G_sol_ (PP) (3)

The bound ligand surroundings include the part that is in contact with the protein (described by V_int_ (PP* − Aβ*)) and the part that is exposed to solvent (described by G_sol_ (PP*Aβ*)). The G_sol_ terms are derived from the GROMOS-calculated solvent interaction energies, V_sol_, after application of a modification of linear response theory [65],

G_sol_ (PP) = βV_sol,es_ (PP) + αV_sol,vdW_ (PP) (4)

and

G_sol_ (PP*Aβ*) = βV_sol,es_ (PP*Aβ*) + αV_sol,vdW_ (PP*Aβ*) (5)

where PP* indicates the PP* moiety in PP*Aβ* (not Aβ*, which is assumed to cancel).

In addition, the electrostatic and van der Waals parts of the interaction energy are also separated:

V_int_ (PP* − Aβ*) = βV_int,es_ (PP* − Aβ*) + αV_int,vdw_ (PP* − Aβ*) (6)

The LIE-S β and α are parameters with values β = 0.5, α = 0.161. Thus, the electrostatic and van der Waals parts of the interaction of the ligand with solvent and protein are scaled differently and contributions from the protein are assumed to cancel.

∆G_binding_^LIE-S^ ≈ β (V_int,es_ (PP* − Aβ*) + V_sol,es_ (PP*Aβ*) – V_sol,es_ (PP)) + α (V_int,vdw_ (PP* − Aβ*) + V_sol,vdw_ (PP*Aβ*) – V_sol,vdw_ (PP)) + γ (7)

The parameter, γ, is potentially a fitting constant which in LIE-S is taken as γ = 0. A more accurate version of LIE-S has been suggested, in which the parameters α and β were fitted to a training set of free energy perturbation-derived energies. A constant value of α = 0.18 [66] was adopted and it was found that an improved fit could be obtained if the value, β = 0.43 [66], was adjusted up or down to account for the different functional groups in the ligand [67].

A further improvement to the LIE-S procedure, coined LIE-D, has been proposed [68], based on a modification of Equation (7) to include electrostatic intra-ligand terms, V_gas,es_ (PP*Aβ*) and V_gas,es_ (PP):

∆G_binding_^LIE-D^ ≈ β (V_gas,es_ (PP*Aβ*) + V_int,es_ (PP* − Aβ*) + V_sol,es_ (PP*Aβ*) − V_gas,es_ (PP) − V_sol,es_ (PP)) + α (V_sol,vdw_ (PP*Aβ*) + V_int,vdw_ (PP* − Aβ*) − V_sol,vdw_ (PP)) + γ (8)

where PP*Aβ* indicates that only the “PP*” moiety of the bound complex is used and PP is the unbound ligand. The intraligand van der Waals terms are not included in Equation (8). The α and β parameters are the same as proposed by Almlof, et al. [67]. The γ parameter is obtained assuming a proportionality between it and a new parameter, D, that accounts for the difference between the polar and nonpolar contributions to the binding free energy of the ligand.

D = β (V_gas,es_ (PP*Aβ*) + V_int,es_ (PP* − Aβ*) + V_sol,es_ (PP*Aβ*) − V_gas,es_ (PP) − V_sol,es_ (PP)) – α (V_sol,vdw_ (PP*Aβ*) + V_int,vdw_ (PP* − Aβ*) − V_sol,vdw_ (PP)) (9)

and

γ = f × D + g(10)

where f = −0.95 and g = −2.06 (kcal/mol) from a training set of 24 protein ligand complexes [68]. The parameter, α, has the value α = 0.18. The parameter β is adjusted from β = 0.43 on the basis of the functional groups present according to the prescription in Almlof, et al. [67].

In the present case, the approximation that the “protein,” that is, Aβ_42_, is rigid is not justified. We propose a further improvement to the LIE-D procedure, LIE-DR, to at least partially correct this deficiency. If one were simply to reverse the roles of ligand and protein in Equations (8) and (9), one obtains:

∆G_binding_^LIE-DR^ ≈ β (V_bond,es_(PP*Aβ*) + V_int,es_ (PP* − Aβ*) + V_sol,es_ (PP*Aβ*) − V_bond,es_ (Aβ) − V_sol,es_ (Aβ)) + α (V_sol,vdw_ (PP*Aβ*) + V_int,vdw_ (PP* − Aβ*) − V_sol,vdw_ (Aβ)) + γ (11)

This reversal of roles has two important justifications in the present context:

The primary interest of the present study is not to monitor a change in the ligand (PP) but in the protein (Aβ_42_). The two ligands were specifically designed to bind to Aβ and restrict its conformational space so as to hinder aggregation through β-sheet formation.

Aβ is far more flexible than the pseudo-peptides, SGB1 and SGD1. Equation (11) should provide a better estimate of the relative energies of conformations of the same PPAβ system and a better estimate of the relative binding affinities of the two ligands.

Having no better option, we have adopted the literature values of α, β and γ for Equations (8) and (11). Results for the Aβ-SGB1 and Aβ-SGD1 systems for the Gas-PBSA, LIE-D and LIE-DR methods are listed in result.

The terms for PP and Aβ correspond to individually equilibrated structures and are treated in the same way as the PP-Aβ clusters. G_gas_ (Aβ) = 4376 kJ/mol was reported previously [66]. The electrostatic and van der Waals components are V_gas,es_ (Aβ) = 5239 kJ/mol and V_gas,vdW_ (Aβ) = −863 kJ/mol. The PBSA value, G_sol_ (Aβ) = −1915 kJ/mol, compared to the PCM value for the central structure, −1949 kJ/mol [66]. Error estimates for the quantities in Equation (2) were derived by the block-averaging procedure described by Hess [69].

## 3. Results for SGB1, SGD1, SGB1-R and SGD1-R

### 3.1. The Monomers

The MD simulation of R as previously reported [57], produced a major cluster with a population of 63%. This central conformer possesses a hairpin turn and a concomitant intramolecular β-sheet. On the other hand, the populations of the first clusters of SGB1 and SGD1 were 87% and 75%, respectively. Comparing these with R, both pseudo-peptides are less flexible than R and possess an extended beta-strand backbone conformation (Figure 1a,b). The additional backbone rigidity may be attributable to the inserted *N*-methylation. For RMSD and scatter plot analyses, see Supplementary Materials.

### 3.2. The Binary Complexes, SGB1-R, SGD1-R, SGB1-SGB1 and SGD1-SGD1

**Figure 2:**
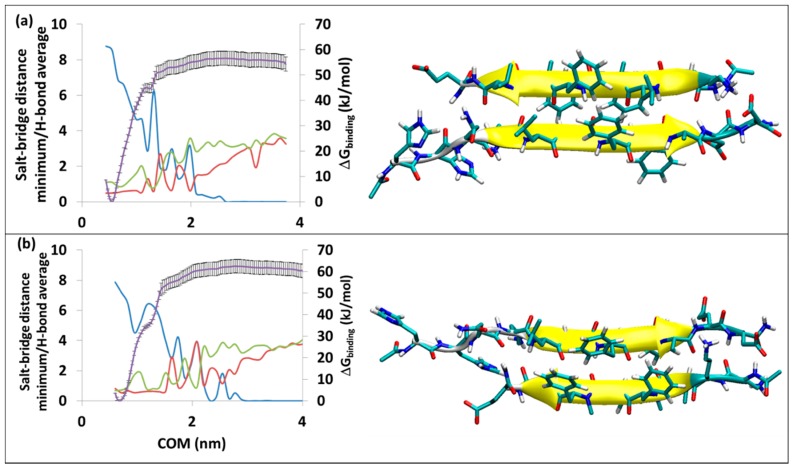
Binding of SGB1 to R at (**a**) R^T^-SGB1, on the top edge, (**b**) R^B^-SGB1, on the bottom edge. The right-hand axis is for PMF curve (purple line) in kJ/mol, the error bars are included (±1σ). The left-hand axis has two functions: a numerical count of the average intermolecular H-bonds (blue line) and a distance measure in nm for the separations of the polar charged residues (salt-bridges), Lys16-glu8′ (green line) and minimum distances for Glu22-daba1′, Glu22-orn2′, Asp23-daba1′, Asp23-orn2′ (red line). The horizontal axis is the separation of the centers of mass in nm.

**Figure 3:**
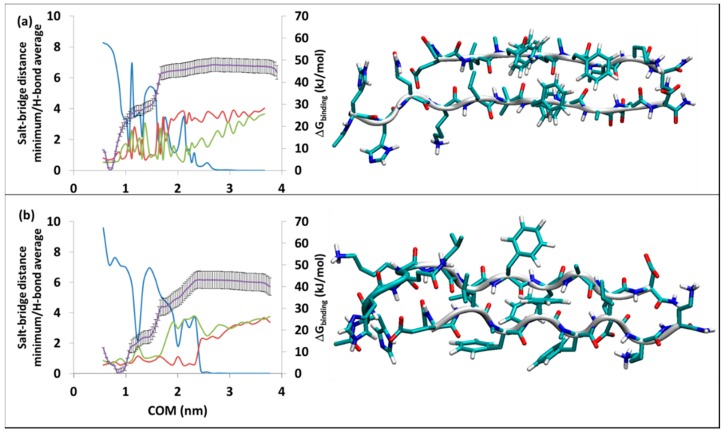
Binding of SGD1 to R at (**a**) R^T^-SGD1, on the top, (**b**) R^B^-SGD1, in the bottom. The right-hand axis is for PMF curve (purple line) in kJ/mol, the error bars are included (±1σ). The left-hand axis is the average intermolecular H-bonds (blue line) and salt-bridge distances at the polar charged residues in nm; Lys16-glu8′ (green line) and minimum distances for Glu22-daba1′, Glu22-orn2′, Asp23-daba1′, Asp23-orn2′ (red line). The horizontal axis is the separation of the centers of mass in nm.

As with the all-l-amino acid pseudo-peptides of the SGA and SGC series previously discussed [30,31], the all-d-pseudo-peptides, SGB1 and SGD1, form antiparallel and parallel β-sheet structures, respectively, when bound to R. There are two possibilities for either SGB1 or SGD1 to interact with the β-strand of R peptide. With the standard positioning of R, the pseudo-peptides can be introduced either on the top edge (T-edge) or on the bottom edge (B-edge) of R. The resulting structures are shown in Figure 2 and Figure 3 for R^T^-SGB1, R^B^-SGB1 and R^T^-SGD1, R^B^-SGD1, respectively. These structures are representative of the first cluster, that is, the cluster with the highest population. For the corresponding scatter plots (evolution of conformers as a function of time) and RMSD calculation, refer to Supporting information. The registry (where applicable), population, number of H-bonds and free energy of binding, ∆G_binding_, are listed in Table 1.

#### 3.2.1. The R^T^-SGB1 and R^B^-SGB1

The stability and structural data on R^T^-SGB1 and R^B^-SGB1 are in Table 1. MD simulation of R^T^-SGB1 was carried out with a starting structure having the registry of *i + j =* 23; where *i* is from the numbering of R (=Aβ_13–23_) and *j* is the natural numbering of the pseudo-peptides. The first highly populated cluster of R^T^-SGB1 (Figure 2a) had a population of 99% during a 50 ns MD simulation, which indicates high rigidity. In the case of R^B^-SGB1, the registry of *i + j =* 24. The first cluster for R^B^-SGB1 (Figure 2b) had a population of 89%, in the 50 ns time of MD simulation. Both complexes are antiparallel β-sheets, as shown in Figure 2.

The free energy of binding from MD-US is ∆G_binding_ = −57 ± 3 and ∆G_binding_ = −62 ± 3 kJ/mol for R^T^-SGB1 and R^B^-SGB1, respectively. Data for some of the all-l-ligand complexes with R, are presented in the lower half of Table 1. It is evident that SGB1 binds as strongly, or more strongly, to R than any of the all-l-pseudo-peptides.

The average number of hydrogen bonds in R^T^-SGB1 is 8.8 (the blue line in Figure 2a). As the separation of COM increases, it decreases to 2.5 before rising briefly to 6.2 at COM of 1.3 nm. This incidence makes a small ridge at the PMF curve (the purple line) at the COM of 1.3 nm. The hydrogen bonds vanish at a COM separation of 2.8 nm. This point is where the salt bridges at both sides of the complex (the green and red lines) disappear, the peptides are completely separated and the PMF curve plateaus.

The average number of hydrogen bonds in R^B^-SGB1 is 7.9. During the pulling process, the hydrogen bonds are broken and sometimes built again but after a COM separation of 2.90 nm, all of the H-bonds disappear. The C-terminal residues of R (Glu22 and Asp23) maintain a salt bridge with the N-terminal residues of SGB1 (daba1, orn2), until a separation of 2.65 nm (red line). On the other side of β-sheet, Lys16 of R is not strongly associated with glu8 of SGB1 (green line). After the complete separation of the peptides, the plateau in the PMF curve (purple line in Figure 2b) appears.

#### 3.2.2. The R^T^-SGD1 and R^B^-SGD1

The stability and structural data for R^T^-SGD1 and R^B^-SGD1 are given in Table 1. Details on the umbrella sampling and the most stable structures are provided in Figure 3a,b, respectively. The all-d-amino acid SGD1 is designed to bind to R in parallel β-sheet fashion. VMD detected the most stable structures to be a pair of parallel β-strands (Figure 3a,b), visually the structures appear to be parallel β-sheets.

For R^T^-SGD1, the first cluster persisted through the whole time of 150 ns of equilibration, with a population of 73%. Based on the PMF curve in Figure 3a, R^T^-SGD1 has the ∆G_binding_ of −50 ± 3 kJ/mol. The average number of intermolecular hydrogen bond is 8.3 at the beginning of the simulation. As the separation increases, this value oscillates till a COM separation = 2.8 nm after which there are no hydrogen bonds. Similar to other cases, the average salt-bridge distances are in accordance with the hydrogen bond counts. For Lys16-glu1′, the COM of 1.7 nm, is the last point that the salt-bridge with the distance of 1 nm is observed. After this point, the salt-bridge is completely gone. On the other hand, the Glu22-orn7′ and Asp23-daba8 counterparts persist slightly further in the reaction coordinate, till the COM of 2.2 nm, where the salt-bridge disappears.

The R^B^-SGD1 complex was equilibrated for 400 ns and the first cluster through the course of the trajectory appeared after 150 ns and existed for the rest of 400 ns. Based on RMSD calculation and cluster analysis, a cluster analysis for the last 250 ns was performed; the result showed a population of 93% for this cluster through the equilibrated time, with the average number of intermolecular hydrogen bonds of 9.6 (the blue line). The high initial hydrogen bond count within the complex suggests a high ∆G_binding_. However, the PMF curve from umbrella sampling calculation estimated ∆G_binding_ = −43 ± 4 kJ/mol (purple line in Figure 3b) which is lower than the case of R^T^-SGD1. The green line stands for the average distance of salt-bridges between Lys16 and glu1′. Glu22 and Asp23 make salt-bridges with orn7′ and daba8′ shown in red line in the Figure 3b. After a COM separation of 2.4 nm, all the interactions disappear and the plateau on the PMF curve appears.

#### 3.2.3. SGB1 and SGD1 Homodimers

**Figure 4:**
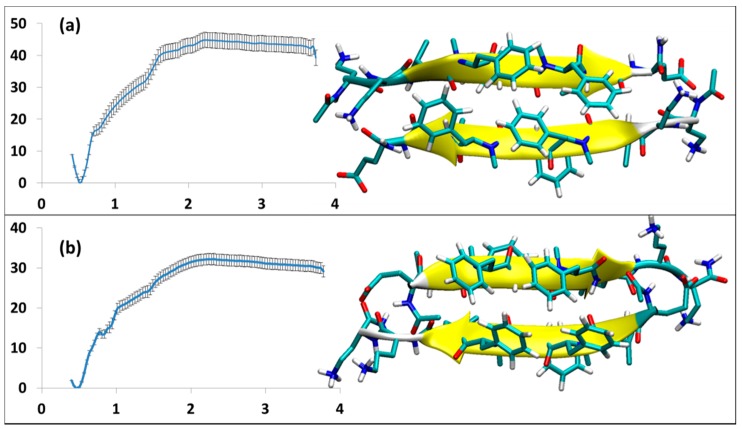
The homodimers; the structures and the related PMF curve: (**a**) SGB1-self (**b**) SGD1-self.

The structures and energy analysis data for the homodimers of SGB1 and SGD1 are shown in Figure 4a,b, respectively. Both form antiparallel β-sheets. The registry of the dimers is 10 and 8 for SGB1 and SGD1 homodimers, respectively. The SGB1 homodimer has an average number of 6.6 intermolecular H-bonds. It had a population of 94% during equilibration of the 50 ns MD simulation. The high population suggests that the SGB1-SGB1 structure is very rigid. The binding energy is relatively high, ∆G_binding_ = −45 ± 3 kJ/mol. The 50 ns MD simulation for SGD1-SGD1, produced a structure with 80% population, with the average number of intermolecular H-bonds of 7.1. For SGD1-SGD1, ∆G_binding_ = −32 ± 2 kJ/mol.

### 3.3. The Effective ∆G_eff_

The quantity, ΔG_eff_, is defined as the free energy change for the reaction,

PP-PP + R-R → 2 R-PP ∆G_eff_ (12)

It is a measure of whether the pseudo-peptide, PP, is likely to dissociate a dimer of Aβ, modelled as R-R, or conversely, whether the complex, PP-Aβ, will prevent the first step in the aggregation of Aβ. ΔG_eff_ is readily calculated from the ΔG_binding_ values from MD-US listed in Table 1. SGB1 is predicted to be particularly effective in this regard, with ΔG_eff_ values of −16 kJ/mol and −26 kJ/mol for the R^T^-SGB1 and R^B^-SGB1 complexes, respectively. On the other hand, SGD1 is predicted to be less effective: ΔG_eff_ = −15 kJ/mol and −1 kJ/mol for the R^T^-SGD1 and R^B^-SGD1, respectively.

In summary, we have studied four classes of pseudo-peptides as described in the introduction and the energy data relevant to the top pseudo-peptide in each class are gathered in Table 1. Among this list, the pseudo-peptide with the most negative ∆G_eff_ is the all-l-amino acid SGC1 and then all-d-amino acid SGB1. The large absolute value stems from fact that the SGB1 has strong binding to R at both edges, with the values of ∆G_binding_ = −57 and −62 kJ/mol. With the B-edge more strongly bound than the T-edge, it is expected that more complexes will be formed in the R^B^-SGB1 form than the R^T^-SGB1 form.

The reversed all d-amino acid pseudo-peptide, SGD1, has negative values for ∆G_eff_, as well, ∆G_eff_^RT-SGD1^ = −15 kJ/mol and ∆G_eff_^RB-SGD1^ = −1 kJ/mol, although not as high as SGB1 and SGC1. The lower ∆G_eff_ in SGD1, stems from the lower ∆G_binding_. Specifically, B-edge of R-SGD1 has lower ∆G_binding_ = −43 ± 4 kJ/mol, compared to values from SGB1 and SGC1, which affects the ∆G_eff_.

## 4. Results for Aβ_42_-SGB1 and Aβ_42_-SGD1

**Figure 5:**
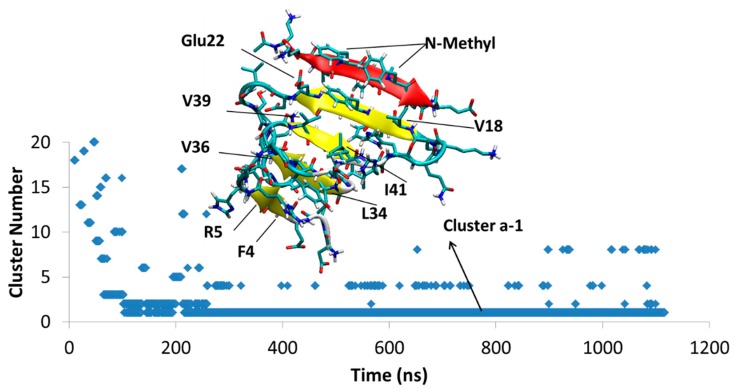
Aβ_42_^T^-SGB1-a; The cluster Ba1 structure is shown, which persist through almost 1 μs. The vertical axis is the cluster number count based on population, where cluster 1 has the highest population. The horizontal axis is simulation time in nanoseconds.

**Figure 6:**
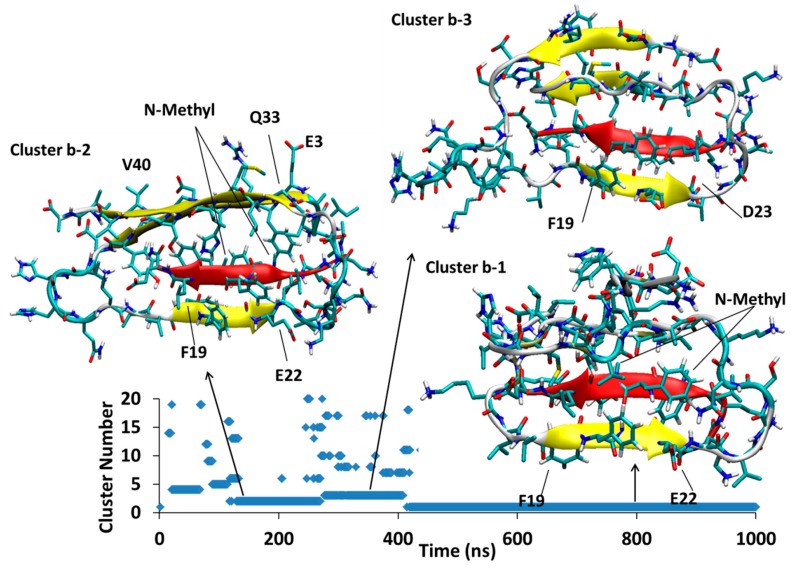
Aβ_42_^B^-SGB1-b; Three major clusters are depicted; Cluster Bb1, Bb2 and Bb3. The vertical axis is the cluster number count based on population, where cluster 1 has the highest population. The horizontal axis is simulation time in nanoseconds.

**Figure 7:**
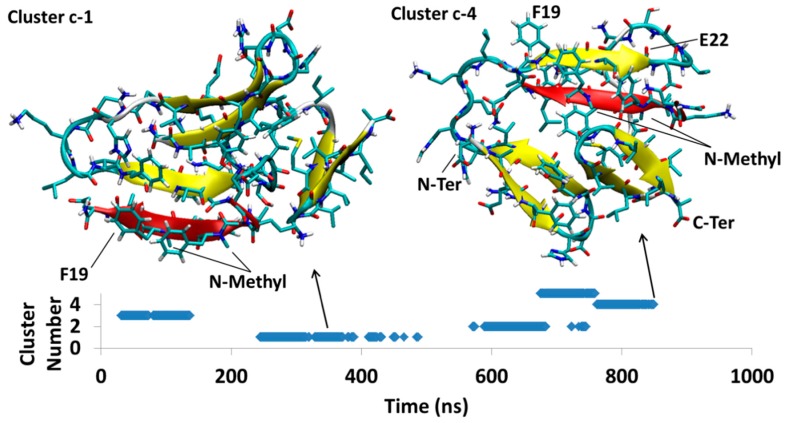
Aβ_42_^B^-SGB1-c; cluster Bc1 with the highest population of similar conformations and Bc4, the last significant cluster in the trajectory are shown. The vertical axis is the cluster number count based on population, where cluster 1 has the highest population. The horizontal axis is simulation time in nanoseconds.

**Figure 8:**
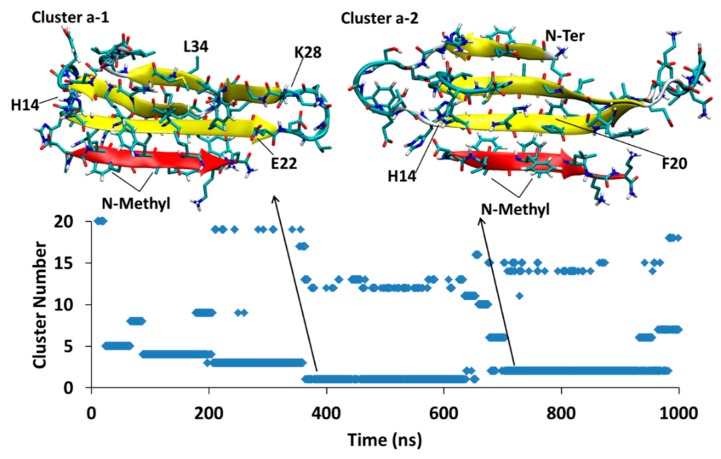
Aβ_42_^T^-SGD1-a: represented the two most populated clusters; cluster Da1 and cluster Da2 in the trajectory.

**Figure 9:**
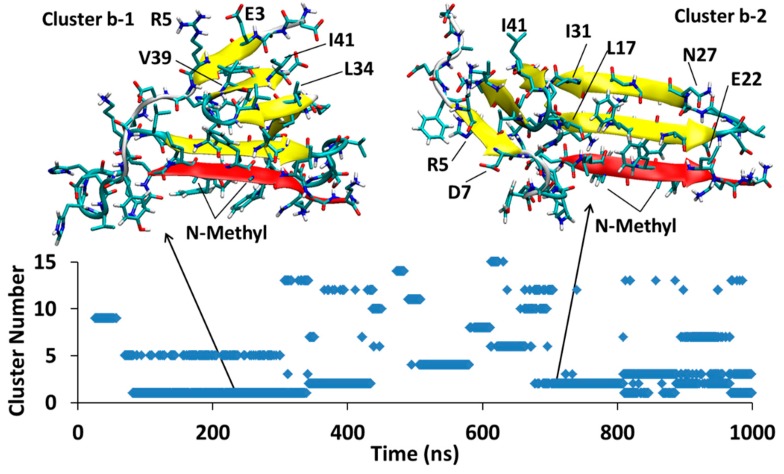
Aβ_42_^B^-SGD1-b: The two ensembles with the highest population are shown. Both Cluster Db1 and cluster Db2 disappear in the middle of trajectory but return for the last 300 and 200 ns of simulation, respectively.

**Figure 10:**
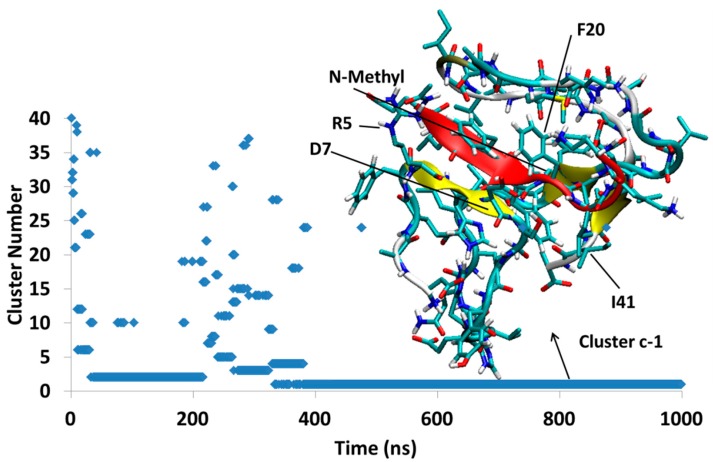
Aβ_42_-SGD1-c: The first cluster, Dc1, exists almost through all of trajectory, with no β-sheet with R region. The total number of clusters through 1 μs was only 40.

**Figure 11:**
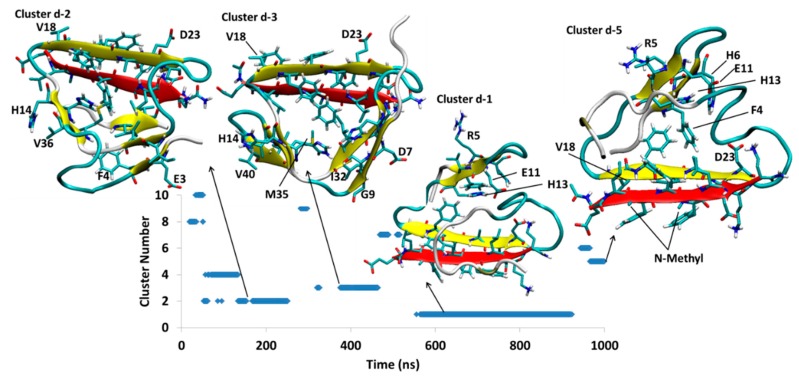
Aβ^B^-SGD1-d. Clusters Dd1, Dd2, Dd3 and Dd5 are shown.

MD of each system was initiated from several different docking poses of SGB1 and SGD1 with the extended structure of Aβ shown in Figure 1d. These are identified with lower case letters a, b, c and so forth. After a suitable length of simulation time, typically 1000 ns, cluster analysis was performed. The clusters were numbered according to their populations by Arabic numerals, 1 (highest), 2, 3 and so forth. For example, the full label, Aβ_42_-SGB1-b-3, which we also shorten to Bb3, corresponds to the third-most populated cluster of the simulation started from the second docked pose of Aβ-SGB1. The evolution of clusters during the 1000 ns trajectory is described by a scatter plot. Scatter plots are shown for all PP-Aβ simulations in Figure 5, Figure 6, Figure 7, Figure 8, Figure 9, Figure 10 and Figure 11. As each simulation is far from equilibrium, no significance is attached to the cluster populations, unlike for R-PP. Special attention is given to clusters that survive toward the end of the simulation as these should be lower in energy than clusters that appeared early in the simulation and did *not* reappear toward the end. For each significant cluster, energy analyses, Gas-PBSA, LIE-D and LIE-DR, were performed as described in Methods as a more reliable means of assigning relative energies of clusters within a simulation and across simulations.

### 4.1. Aβ_42_-SGB1

Three MD runs with different starting structures have been studied for Aβ-SGB1 complex and named as Ba, Bb and Bc. In Ba, SGB1 is attached to the top “T” edge of the R region of Aβ, while for Bb and Bc, attachment is to the bottom edge.

#### 4.1.1. Aβ^T^-SGB1-a

After the first 103 ns, a single structure, Cluster Ba1, persists through the rest of the 1.1 μs duration of the simulation. The Aβ part of the structure has a four β-sheets. SGB1 is attached to the “T” edge of the outermost β-strand forming an antiparallel β-sheet between leu3–leu7 from SGB1 and Val18-Glu22 from Aβ; the *N*-methylated groups are located outside of the β-sheet and exposed to the aqueous environment. The registry of this β-sheet is *i* + *j* = 25, where *i* is from the Aβ_1–42_ numbering of R and *j* is the natural numbering of the pseudo-peptide. Thus, the attachment is in the R region of Aβ. The Aβ^T^-SGB1 complex can be compared with R^T^-SGB1 discussed above, for which the registry was 23. There are intramolecular antiparallel and parallel β-sheets in the Aβ moiety, as shown in Figure 5.

#### 4.1.2. Aβ^B^-SGB1-b

In Figure 6, the Aβ^B^-SGB1-b complex has three major clusters, Bb1, Bb2 and Bb3, which appear in the order Bb2 < Bb3 < Bb1; the registry for all of these clusters is *i* + *j* = 26, where *i* is from the Aβ_1–42_ numbering of R and *j* is the natural numbering of SGB1. For all the clusters, there are salt-bridges between Glu22 and Asp23 to Orn2′ and Asp23 to Dab1′. These residues are not in front of each other when considering the registry but they bend to reach each other to make such interactions.

Between 118–267 ns, a significant cluster with the second highest population exists and is designated as cluster Bb2. The residues Glu3-Tyr10 (N-Terminal region) and Gly33-Val40 (C-Terminal region) form an antiparallel β-sheet in Aβ structure. Residues Phe19-Glu22 and mephe4-leu7 make an antiparallel β-sheet with the “B” edge of Phe19-Glu22 of Aβ.

At 276 ns, Bb2 has converted to Bb3, which persists until 400 ns into the simulation before it converts to Bb1. Cluster Bb3 has two antiparallel β-sheets; one in Aβ between Val36-Gly33 with Glu3-His6 and another at Phe20-Asp23 with leu3-mephe6 of SGB1

At 400 ns, Bb3 converts to Bb1 which exists for the remainder of the 1 μs simulation. The intramolecular β-sheet in cluster Bb3 is not present in cluster Bb1, which has only one antiparallel β-sheet interaction between the “B” edge of the strand F19-E22 (part of the R region) and mephe4-leu7 (from SGB1).

#### 4.1.3. Aβ^B^-SGB1-c

For Aβ^B^-SGB1-c complex no long-lived clusters were observed in the course of the 1 μs simulation. However, there are multiple conformational changes occurring through the Aβ peptide backbone, where at the midpoint of the simulation, the Aβ strand attached to SGB1 flips inside out and wraps around SGB1 (in red in Figure 7). This places the *N*-methyl groups of mephe4 and mephe6 inside Aβ, shielding them from the aqueous environment. We illustrate the flipping process by focusing on two of the clusters, Bc1, that existed between 244 ns and 487 ns and had the highest population and Bc4 which was the last cluster in the simulation. The intermolecular salt-bridges are only observed within the residues Asp23-Orn1 and Asp23-Dab1. The intermolecular β-sheet is very similar to the one in the SGB1-Aβ_42_-b with the same registry of *i + j* = 26. The intermolecular β-sheet is between Phe19-Glu22 and mephe4′-leu7′ and the intramolecular β-sheets are within Ala2-Arg5 with Gly9-Val12 and also Leu34-Val36 and Val39-Ile41.

#### 4.1.4. Relative Energies of the Aβ42-SGB1 Complexes

The results from three schemes, Gas-PBSA, LIE-D and LIE-DR, for the estimation of relative free energies of the Aβ_42_-SGB1 complexes are provided in Table 2. Shortly after the beginning of the MD simulation of Aβ_42_-SGB1-a (Figure 5), a single cluster dominated most of the 1000 ns. Estimates of the energy of binding for this cluster, according to Gas-PBSA, LIE-D and LIE-DR are ΔG_binding_ ≈ +78 kJ/mol, −33 kJ/mol and −26 kJ/mol, respectively (Table 2). We note here that due to the approximate nature of the energy estimates, not much significance can be placed on the absolute magnitudes of the calculated ΔG_binding_ values but clearly, the Gas-PBSA and the LIE-based methods differ widely for the complex, Ba1. In Ba1, SGB1 is bound to the “T” edge of the R-region of Aβ. The more accurate MD-US value for R^T^-SGB1 is −57 ± 3 kJ/mol (Table 1). Although the registries are different, *i* + *j* = 25 in the case of Ba1 and *i* + *j* = 23 for R^T^-SGB1, the comparison suggests that the LIE values may be more accurate.

The same approximations for energy estimates apply to all the PP-Aβ complexes in Table 2, so we expect the relative energies will be more accurate than the absolute values. The second MD simulation of Aβ-SGB1, namely Aβ-SGB1-b, yielded three dominant clusters (Figure 6), in order of appearance, Bb2, Bb3 and Bb1. According to LIE-D and LIE-DR, the energies of the complexes were the same within the error bars: by LIE-D, −58 kJ/mol (Bb2), −67 kJ/mol (Bb3) and −64 kJ/mol (Bb1); by LIE-DR, −102 kJ/mol (Bb2), −106 kJ/mol (Bb3) and −101 kJ/mol (Bb1). Especially by LIE-DR which more accurately accounts for the flexibility of the Aβ moiety, the Bb simulation has yielded more stable structures than the Ba simulation despite the apparent stability of Ba1. The same is true of the Gas-PBSA estimates. The reason for the greater stability of the “Bb” series and especially Bb1, over Ba1, can be attributed to a significantly greater interaction energy, V_int_ (SGB1* − Aβ*), namely −668 ± 7 kJ/mol versus −349 ± 5 kJ/mol, respectively (Table 2).

In the Bb series, SGB1 is attached to the “B” edge of the R region of Aβ and comparison can be made with the MD-US results for R^B^-SGB1 for which ΔG_binding_ ≈ −62 ± 3 kJ/mol (Table 1). Thus R^B^-SGB1 is marginally more stable than R^T^-SGB1. The equivalent attachment of SGB1 to full length Aβ yields structures, Bb, which are substantially more stable than Ba1 (LIE-DR values), ΔG_binding_ ≈ −105 ± 41 kJ/mol versus ΔG_binding_ ≈ −26 ± 41 kJ/mol, respectively, despite the large error bars. We note here again that the registries are different, *i* + *j* = 26 and, *i* + *j* = 24, for Bb and R^B^-SGB1, respectively.

The third simulation, Aβ-SGB1-c (Figure 7) did not yield a single dominant structure during the 1000 ns simulation. The three energy schemes are in agreement that the last structure Bc4 is more stable than the first, Bc1 but the Gas-PBSA and LIE schemes yield very different results in an absolute sense, as they did in the case of Ba1. According to LIE-D and LIR-DR, structure Bc4 has similar stability to the Bb structures. As in the case of Bb, Bc4 has “B” edge attachment of SBG1, with registry, *i* + *j* = 26 and a large interaction energy, V_int_ (SGB1* − Aβ*) = −567 ± 8 kJ/mol.

### 4.2. Aβ_42_-SGD1

SGD1 is designed to make a parallel β-sheet with the R region of Aβ. There are four different simulations from four different starting structures for the Aβ-SGD1 system; a top edge interaction of the R region of Aβ with SGD1 (Aβ^T^-SGD1-a), a bottom edge interaction of R with SGD1 for Aβ^B^-SGD1-b, a structure, Aβ-SGD1-c, not involving the R region and finally a structure, Aβ^B^-SGD1-d, that was mutated from Aβ-SGB1-b series. Each simulation was run for 1 μs.

#### 4.2.1. Aβ^T^-SGD1-a

The simulation for Aβ_42_^T^-SGD1-a is shown in Figure 8. There are two major clusters that exist in the 1 μs time of the simulation, cluster1-a (Da1, through 364–650 ns) and cluster2-a (Da2, through 638–977 ns). In both clusters, Aβ_42_-SGD1 is has extensive parallel β-sheet character. For cluster Da1, all the amino acids in SGD1 are involved in the created β-sheet, that is, glu1′-daba8’ with His14-Ala21. For cluster Da2, there’s a shorter intermolecular β-sheet, within glu1′-leu6′ with His14-Phe19.

#### 4.2.2. Aβ^B^-SGD1-b

The simulation for Aβ_42_^B^-SGD1-b is shown in Figure 9. Two major clusters appear in the trajectory. Cluster Db1 emerges in 171–340 ns and then disappears but reappears intermittently at 810 ns and stays till the end of simulation time. Likewise, cluster Db2 exists at 342–435 and then comes back between 680 ns and 967 ns. Both structures have parallel β-sheet interaction between the SGD1 (within leu2′-leu6′) with R region of Aβ (Val18-Glu22). This β-sheet interaction is in such way that all the possible salt-bridges within the two monomers are made. The intramolecular β-sheet interactions are very similar for both clusters and they are within Leu34-Val36 (the second hydrophobic region) and Val39-Ala42 (C-terminal) and the N-Terminal region of Aβ.

#### 4.2.3. Aβ-SGD1-c

The simulation, Aβ-SGD1-c, was started from an unstable docked structure in which the *N*-methyl groups were directed toward the R region of Aβ. Within a few ns after the start of the simulation, SGD1 separates from the R region and after about 320 ns settles into a structure, Dc1, that lasts for the duration of the 1000 ns. In Dc1, the SGD1 peptide interacts through its residues leu2′-phe4′, to form a short β-sheet with Arg5-Asp7 of Aβ (Figure 10).

#### 4.2.4. Aβ^B^-SGD1-d

The initial structure for Aβ^B^-SGD1-d was obtained by mutation of SGB1 in Aβ-SGB1-b into SGD1. Figure 11 shows three clusters, Dd2, Dd3 and Dd1 with moderately high populations and small cluster, Dd5, which existed for the last 50 ns of the simulation. Cluster Dd2, exists at the time interval of 53–251 ns. In this structure, SGD1 within leu2′-orn7′ is in parallel β-sheet with Val18-Asp23 of Aβ. Val40-Ile41 makes a short intramolecular parallel β-sheet with Phe20-Ala21. The Aβ moiety of Dd2 has other minor intramolecular β-sheets, namely His13-His14 with Met35-Val36 and Glu3-Phe4 with Ile31-Ile32.

Cluster Dd2 makes a transition to structure Dd3 which exists through 321–465 ns. The main intermolecular interaction is similar to cluster Dd2, where the parallel β-sheet is observed between Val18-Asp23 and leu2′-orn7′. This intermolecular interaction is observed also for next clusters, Dd1 and Dd5. In addition to the intermolecular β-sheet interaction, Aβ makes β-sheets at His6-Gly9 with Gly29-Ile32 and also His13-His14 with Met35-Val36 and Val40-Ile41. In the transition from Dd3 to Dd1, the structure goes through a major change, where the intermolecular β-sheet containing SGD1 flips upside down, positioning SGD1 to the outer edge and rearranges its intramolecular β-sheets to the region Phe4-His6 and Glu11-His13. This new structure, Dd1, persists longest through the trajectory. Cluster Dd1 disappears after 923.3 ns. The final structure Dd5 appears to be very similar to Dd1, differing by a rearrangement of the C-terminal region. The population of Dd5 is less than 10% of that of Dd1 at the 1 μs termination of the simulation.

#### 4.2.5. Relative Energies of the Aβ_42_-SGD1 Complexes

The absolute binding free energies for the Aβ-SGD1 complexes are shown in Table 3. The Gas-PBSA and the LIE methods are in agreement that the two structures from the first simulation, Da1 and Da2, are approximately equally stable. The same is true of the two from the second simulation, Db1 and Db2. Both procedures also indicate that Db1 and Db2 are more stable than Da1 and Da2. The Da system and the Db system can be compared with R^T^-SGD1 and R^B^-SGD1, respectively. In the case of the complexes with R, parallel β-sheet binding of SGD1 to the “T” edge was more stable than to the “B” edge by a small margin, −50 ± 3 kJ/mol versus −43 ± 4 kJ/mol (Table 1). The opposite is true for binding to full length Aβ, with the “B” edge being favored over the “T” edge, ≈ −60 ± 41 kJ/mol versus ≈ −40 ± 42 kJ/mol.

There is wide disagreement between Gas-PBSA and the LIE-methods with respect to the stability of the single structure of the third simulation. According to Gas-PBSA, Dc1 is considerably less stable, ΔG_binding_ = 374 kJ/mol, than the Da complexes, ΔG_binding_ ≈ 50 kJ/mol, or the Db complexes, ΔG_binding_ ≈ 13 kJ/mol. On the other hand, LIE-D and LIE-DR yield the opposite results, ΔG_binding_ = −67 kJ/mol and −111 kJ/mol, respectively and predict that Dc1 is more stable than either Da, = −36 kJ/mol and −37 kJ/mol, respectively, or Db, = −48 kJ/mol and −60 kJ/mol, respectively. The anomalously high Gas-PBSA value stems from the low computed G_PBSA_ free energy of solvation, G_PBSA_ = −1971 kJ/mol (Table 3), which is lower than for any of the other complexes in Table 2 and Table 3.

The LIE-D and LIE-DR procedure are in agreement that the stability of the Dd complexes is similar to the Db complexes. Both involve outer edge (“B” edge) parallel β-sheet binding of SGD1 to the R region of Aβ.

## 5. Discussion

The all-d amino acid pseudo-peptides, PP = SGB1 or SGD1, were designed to bind specifically to a section of Aβ peptide, modelled by R, the region spanning residues 13 to 23 [29]. SGB1 binds as an antiparallel β-sheet, SGD1 as a parallel β-sheet. Both have methyl groups on the backbone intended to rigidify the monomer structure as a β-strand and to prevent propagation of the β-sheet when attached to R or Aβ. The peptides were docked to both edges, designated as “T” and “B,” of R and the R region of full-length Aβ and subjected to molecular dynamics simulations of up to 1000 ns duration.

### 5.1. The PP-R Complexes

The stabilities of the PP complexes with R were assessed by the technique of Umbrella Sampling, which yielded moderately accurate relative free energies of binding, Δ*G*_binding_.

Grillo-Bosch et al., discussed different binding interaction criteria in a β-sheet structure [39]. In all-l-amino acid peptides, the orientation of sidechains is such that the sidechains will have in-registry interactions, while for a β-sheet with an all-l strand and an all-d strand, the two sidechains will be on the opposite side of the β-sheet and will not meet each other. If the sidechain-sidechain interactions are sterically repulsive, the all-d peptide should have a higher binding to the all-l Aβ (or R). However, if the interactions are favorable, such as pi-pi stacking, or salt bridge formation, the all-d peptide will be disadvantaged. The Umbrella Sampling calculations suggest that this is true for the retro-inverso PP, SGD1, which binds to R more weakly the all-l amino acid SGC1, or R to itself. However, the all-d PP, SGB1 binds more strongly. The results for SGB1 are in agreement with studies of all-d-amino acid peptides performed by other groups that concluded that d-amino acid versions have similar [32] or higher [37,39,43] propensity towards Aβ than their all-l counterparts.

The same technique was also applied to the dimeric structures, R-R, SGB1-SGB1 and SGD1-SGD1, in order to determine the effectiveness of the pseudo-peptides in preventing aggregation of Aβ, as modeled by the equation,

PP-PP + R-R → 2 R-PP ∆G_eff_

SGB1 had high effective free energy of binding at both edges and a low self-binding. Consequently, the values, ∆G_eff_^RT-SGB1^ = −16 kJ/mol and ∆G_eff_^RB-SGB1^ = −26 kJ/mol (Table 1), indicate a high predicted effectiveness at disrupting β-sheet formation in Aβ.

Previously, the interaction of R with the all-l-amino acid SGC1, with similar sequence as the all-D PP, SGD1, yielded ∆G_eff_^RT-SGC1^ = −27 kJ/mol and ∆G_eff_^RB-SGD1^ = −21 kJ/mol. [31] In the case of SGD1, the umbrella sampling calculations yielded ∆G_eff_^RT-SGD1^ = −15 kJ/mol and ∆G_eff_^RB-SGD1^ = −1 kJ/mol (Table 1). These results suggest that that the retro-inverso all-d-amino acid SGD1 is not as effective at dissociating R as the related retro-inverso all-l-amino acid SGC1 at 1:1 stoichiometry. One notes though, that since the PP are intended to be developed as anti-Alzheimer drugs, one is not limited to stoichiometric quantities and ΔG_eff_ will improve with higher relative concentrations.

### 5.2. The PP-Aβ Complexes

The artifice of using the separation of centers of mass to define a reaction coordinate for Umbrella Sampling is suitable for PP-R complexes since both R and PP are relatively rigid but it is not applicable for PP-Aβ complexes. Instead, the free energy of binding was evaluated by three approximate schemes as described above, Gas-PBSA, LIE-D and LIE-DR. For each method, all of the energy components listed in Table 2 and Table 3 were averaged over the ensemble of all structures that were within RMSD = 0.35 nm of the structures illustrated in Figure 5, Figure 6, Figure 7, Figure 8, Figure 9, Figure 10 and Figure 11. The last, LIE-DR, takes into account the conformational flexibility of the Aβ moiety and should yield the most reliable relative and absolute values for ΔG_binding_. We realize that the simulation times of about 1 μs are, at best, sufficient to locally equilibrate the structures. Eyring rate theory predicts that at T = 310 K, barriers in excess of 40 kJ/mol are unlikely to be crossed during the simulation.

Three simulations were carried out for the SGB1-Aβ_42_ system and four for the SGD1-Aβ_42_ system, each of 1 μs duration, from separate docked structures in which the Aβ moiety was initially stretched out to expose the R region as a β-strand (Figure 1d). In most cases, local equilibration of the Aβ moiety occurred within the first 100 ns of the simulation. With one exception, the PPs, which were docked to the “T” or the “B” edge of the R β-strand, remained in place while the Aβ moiety underwent extensive structural relaxation. In the one exception, SGD1-Aβ-c (Dc, Figure 10), the docking yielded an unstable starting structure in which the backbone *N*-methyl groups were directed toward the R β-strand. This complex dissociated in a little over 200 ns and reattached in a further 100 ns to form a short locally stable β-sheet, not with the R region but with Arg5-Asp7 in the N-terminal region of Aβ. In another simulation of the SGD1-Aβ system, Dd (Figure 11), the initial structure had SGD1 docked to make a β-sheet with the “B” edge of the R region. During the initial 200 ns of reorganization, the system found itself in a complex in which the SGD1 side of the β-sheet was enveloped by a part of the Aβ moiety (Dd2 and Dd3), causing repulsive contacts between the backbone methyl groups and the enveloping Aβ. After about 460 ns, the entire β-sheet with SGD1 still attached to the “B” edge, rotated through 180° to place the methyl groups into a less hindered position (Dd1 and Dd5).

With the substantial reorganizations of both PP-Aβ complexes, it was essential to establish their relative stabilities in order to identify the most stable SGB1-Aβ complex, the most stable SGD1-Aβ complex and to establish which of the PPs binds more strongly to full-length Aβ. We realize that there is no guarantee that either complex represents a global minimum on the multidimensional free energy landscape. The following discussion is based on ΔG_LIE-DR_ values listed in Table 2 and Table 3.

The most stable structure that arose from the three SGB1-Aβ simulations is Bb1 (Figure 6), ΔG_LIE-DR_ ≈ −100 kJ/mol. Bb1 is slightly more stable than Bc4 (Figure 7), ΔG_LIE-DR_ ≈ −90 kJ/mol. In both structures, the SGB1-containing β-sheet is encompassed by the Aβ moiety, which places the backbone *N*-methyl groups into an apparently sterically crowded position. As noted above, when the SGB1 moiety was mutated into SGD1, that is, into the Dd system (Figure 11), the β-sheet turned through 180° so as to relieve the unfavorable interactions. We suspect that had the Bb and Bc simulations been continued for a further microsecond, both Bb1 and Bc4 would have undergone the same transformation into more stable structures. It is significant that while the “Bb”-like structure of Dd, that is, Dd2 or Dd3, transformed into the more stable configuration, Dd1, with ΔG_LIE-DR_ ≈ −75 kJ/mol, in an absolute sense, the “B”-edge bound SGB1 complex is the more stable one, with ΔG_LIE-DR_ ≈ −100 kJ/mol. Intriguingly, the most stable overall complex was found to be Dc1, ΔG_LIE-DR_ ≈ −111 kJ/mol, in which the PP was not bound to the R region at all but rather to the N-terminal region. Logically, a PP specifically designed and optimized to bind to this region will have a higher binding still. However, such a complex may not be effective as an anti-oligomerization agent since the N-terminal region is disorganized in most of the reported fibril structures and not involved in the amyloid-like β-sheet superstructure.

## 6. Conclusions

The β-sheet blocking pseudo-peptides (PP), SGB1 and SGD1, were designed specifically to bind to the central hydrophobic region of Aβ, Aβ_13–23_ (R), with high affinity and to each other, with lower affinity. Evaluation of the free energies of binding by molecular dynamics umbrella sampling established that both should be effective at blocking Aβ oligomerization, as modelled by R, with a slight edge to SGB1. The PP were also docked to the R region of full-length Aβ_42_ and subjected to 7 μs of molecular dynamics simulations, 3 independent simulations of 1 μs duration in the case of SGB1-Aβ_42_ and 4 in the case of SGD1-Aβ_42_. Relative energies were evaluated by schemes based on Poisson-Boltzmann/Surface Area (Gas-PBSA) or variations of Linear Interaction Energy (LIE-D and LIE-DR) approximations. With one exception, the PP remained attached to the R region in the form of antiparallel (SGB1) or parallel (SGD1) β-sheets. The MD results suggest that the preferred position of the PP-incorporated β-sheet is such that the PP is on the outer edge of the complex with its backbone *N*-methyl groups in the less hindered but solvent exposed, orientation. The binding energy analysis indicated that SGB1-Aβ_42_ complex is more tightly bound in the R region than the SGD1 complex. In the one exception, the initial docking aligned SGD1 with its *N*-methyl groups directed toward the R region, an orientation in which β-sheet formation is impossible. The SGD1 was extruded from the R region and became attached instead to the N-terminal region of Aβ. Clearly, full-length Aβ_42_ has many more potential binding sites than just the central core region, R. The most stable of all the complexes examined proved to be the SGD1-Aβ_42_ complex where the SGD1 was not attached to R. Attachment to R is critical to prevent Aβ oligomerization. In this respect, SGB1 may be a better candidate for developing into an anti-Alzheimer’s drug.

## Figures and Tables

**Table 1:** The registry, population (p_i_) and average number of intermolecular hydrogen bonds (H-bond) for the first clusters of R-complexes of pseudo-peptides (the top and bottom edge) and the homodimers, with their corresponding ∆G_dis_, from umbrella sampling.

Complex	Registry ^a^	p_i_	H-Bond ^b^	∆G_binding_ (kJ/mol)	Effective ∆G_eff_^c^ (kJ/mol)
*d* *-amino acid PPs (this work)*					
R^T^-SGB1	23	0.99	8.8	−57 ± 3	−16
R^B^-SGB1	24	0.89	7.9	−62 ± 3	−26
R^T^-SGD1	NA ^f^	0.73		−50 ± 3	−15
R^B^-SGD1	NA ^f^	0.93	9.6	−43 ± 4	−1
SGB1-SGB1	9	0.99		−45 ± 3	
SGD1-SGD1	8	0.80	7.1	−32 ± 2	
*l* *-amino acid PPs:*					
R^B^-R^B d^	38	0.29	10.3	−53 ± 3	-
R^T^-SGA3 ^d^	24	0.35	8.4	−56 ± 3	−3
R^B^-SGA3 ^d^	21	0.31	7.8	−47 ± 2	+5
R^T^-SGC1 ^e^	NA ^f^	0.80	8.1	−53 ± 3	−27
R^B^-SGC1 ^e^	NA ^f^	0.92	8.2	−50 ± 2	−21
SGA3-SGA3 ^d^	10	0.72	-	−46 ± 4	
SGC1-SGC1 ^e^	8	0.97	6.7	−26 ± 3	

^a^ Aβ_42_ numbering of R, natural numbering of the pseudo-peptides; ^b^ The average intermolecular hydrogen bond count from the first cluster in MD simulation; ^c^ The Effective ∆G_eff_ = 2 ∆G_R-PP_ − ∆G_RR_ − ∆G_PP-PP_; ^d,e^ These values were previously reported by Mehrazma et al [30,31]; ^f^ For the parallel β-sheets, the registry does not apply.

**Table 2:** Aβ_42_-SGB1 energy analysis: Aβ-SGB1 (SGB1 = PP) energy analysis (kJ/mol). The clusters are listed by the hierarchy of their appearance in the trajectory ^a^.

Cluster #	P_i_^b^	ΔG_gas-PBSA_^c^	ΔG_LIE-D_^d^	ΔG_LIE-DR_^e^
Ba1	0.8	78 ± 29	−33 ± 4	−26 ± 41
Bb2	0.13	−14 ± 33	−58 ± 7	−102 ± 43
Bb3	0.11	−16 ± 141	−67 ± 4	−106 ± 41
Bb1	0.58	−37 ± 32	−64 ± 4	−101 ± 41
Bc1	0.12	100 ± 29	−33 ± 3	−37 ± 41
Bc4	0.08	50 ± 44	−60 ± 5	−91 ± 41

**^a^** The raw data is in Supporting information Table S2; ^b^ Fractional population; ^c^ Equation (2); ^d^ Equation (8); ^e^ Equation (11).

**Table 3:** Aβ_42_-SGD1 energy analysis: Aβ-SGD1 (SGD1 = PP) energy analysis: (kJ/mol). The clusters are listed by the hierarchy of their appearance in the trajectory ^a^.

Cluster #	P_i_^b^	ΔG_gas-PBSA_^c^	ΔG_LIE-D_^d^	ΔG_LIE-DR_^e^
Da1	0.24	56 ± 30	−37 ± 3	−35 ± 40
Da2	0.23	45 ± 36	−35 ± 3	−40 ± 42
Db1	0.25	16 ± 43	−48 ± 4	−62 ± 41
Db2	0.23	11 ± 62	−47 ± 5	−59 ± 41
Dc1	0.62	374 ± 29	−67 ± 3	−111 ± 40
Dd2	0.09	−58 ± 31	−56 ± 3	−69 ± 41
Dd3	0.08	−41 ± 32	−48 ± 4	−62 ± 44
Dd1	0.33	58 ± 32	−54 ± 3	−75 ± 41
Dd5	0.03	−21 ± 31	−41 ± 3	−42 ± 41

**^a^** The raw data is in Supporting information Table S3; ^b^ Fractional population; ^c^ Equation (2); ^d^ Equation (8); ^e^ Equation (11).

## Supplementary Materials

The Supplementary Materials are available online.
