# Supplementary material for: d-Amino Acid Pseudopeptides as Potential Amyloid-Beta Aggregation Inhibitors
Source: Molecules. 2018 Sep 18;23(9):2387. doi: 10.3390/molecules23092387 (PMC6225248; doi:10.3390/molecules23092387)
Supplement: Supplementary file 1 [file molecules-23-02387-s001.pdf]

Supporting Information for:

D-amino acid pseudopeptides as potential amyloid-beta aggregation inhibitors

B. Mehrazma, S. K. A. Opare, A. Petoyan, A. Rauk*

Department of Chemistry; University of Calgary; Calgary AB, Canada T2N 1N4

*rauk@ucalgary.ca

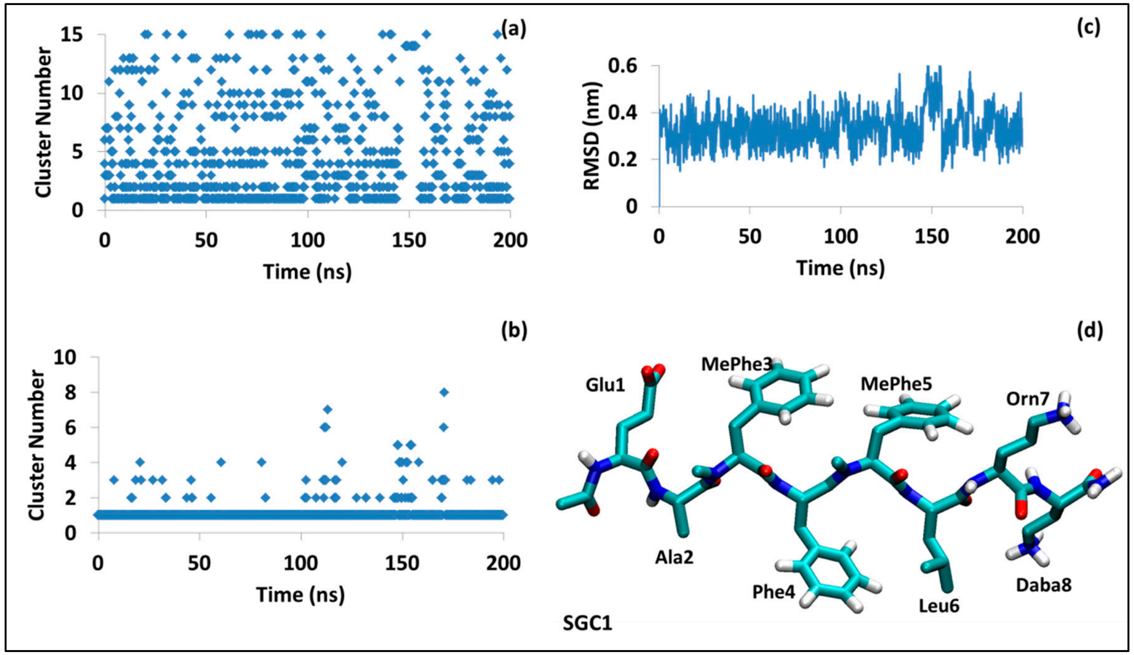

Figure S1 The structure analysis for SGC1, (a) the first 15 clusters for the all-atom based and (b) backbone based cluster analysis; (c) The RMSD calculation profile; (d) the structure of the first cluster of all-atom based cluster analysis.

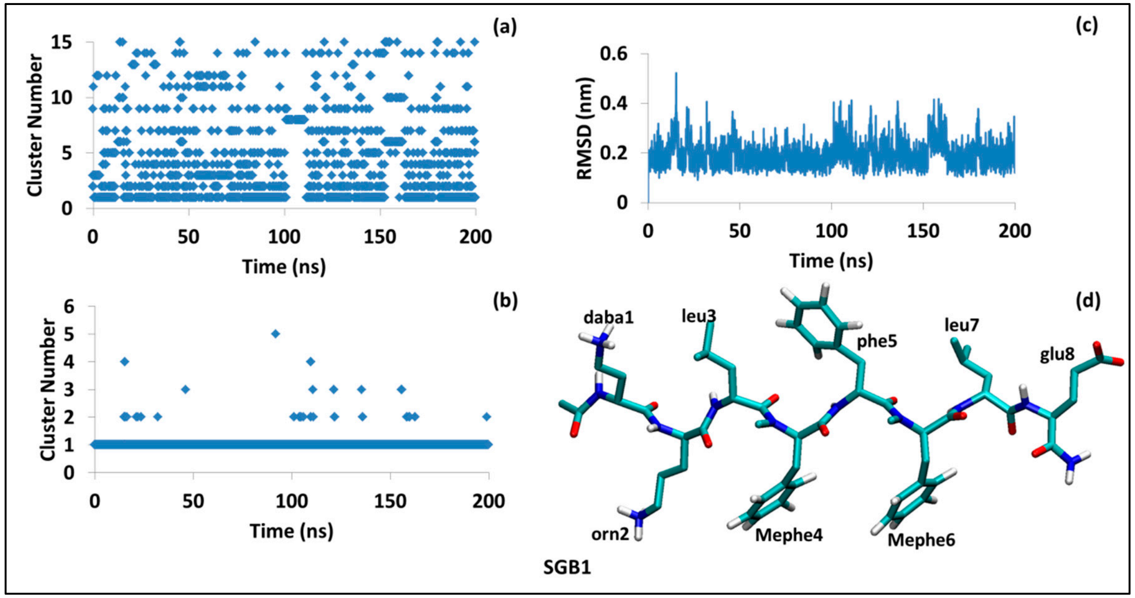

Figure S2 The structure analysis for SGB1, (a) the first 15 clusters for the all-atom based and (b) backbone based cluster analysis; (c) The RMSD calculation profile; (d) the structure of the first cluster of all-atom based cluster analysis.

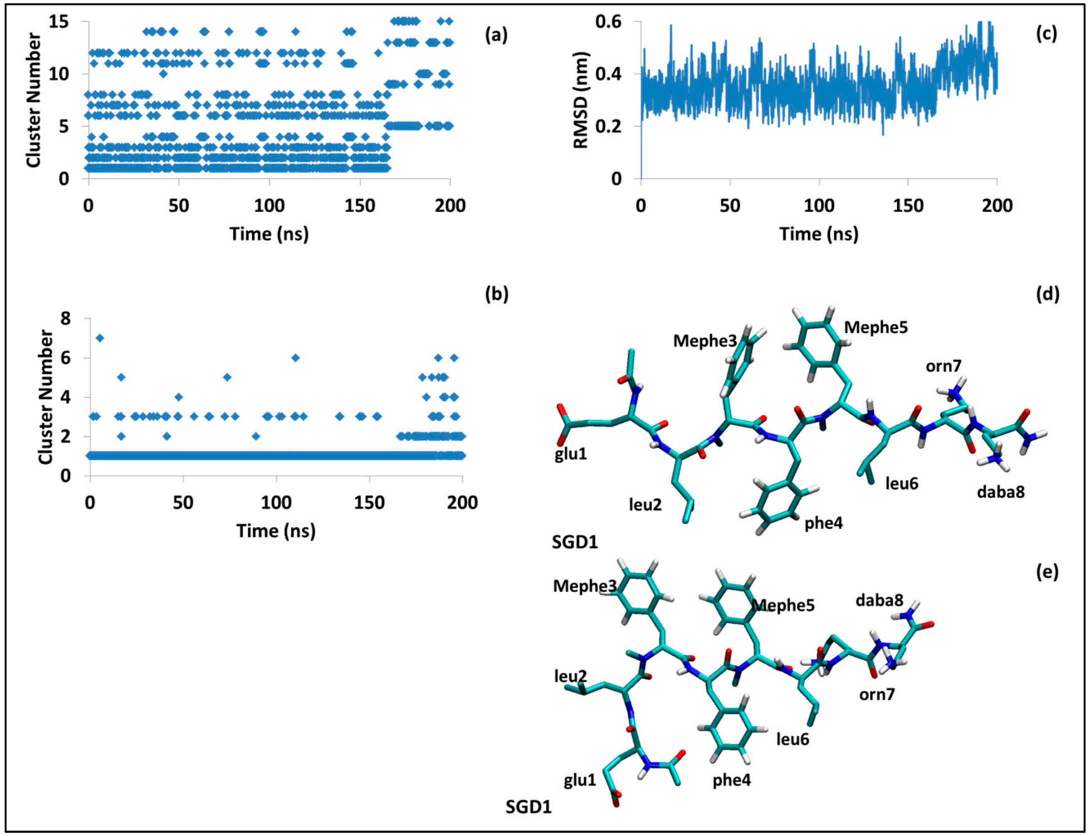

Figure S3 The structure analysis for SGD1, (a) the first 15 clusters for the all-atom based and (b) backbone based cluster analysis; (c) The RMSD calculation profile; (d) the structure of the first cluster and (e) the fifth cluster of all-atom based cluster analysis.

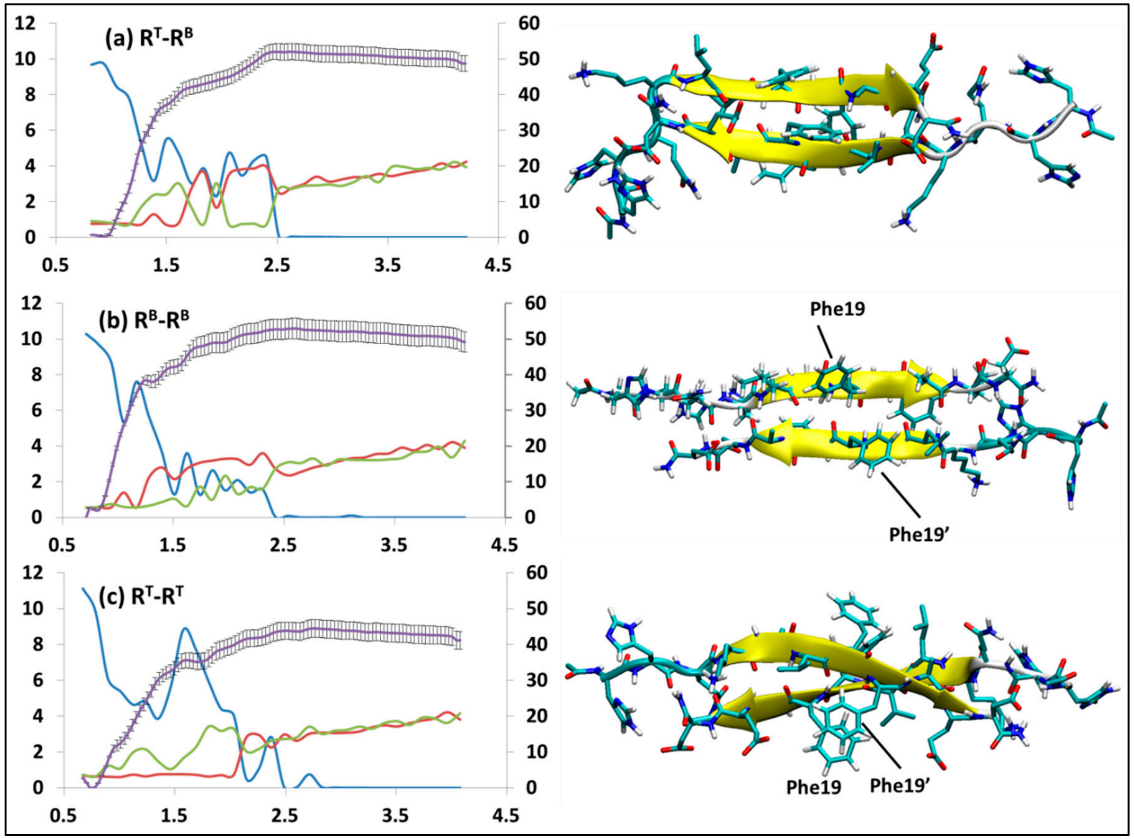

Figure S4 Three binding modes for R defined together with PMF curves. (a) T-edge to B-edge (b) B-edge to B-edge (c) T-edge to T-edge. The right-hand side axis of the graphs refer to PMF curve (kJ/mol) shown in purple line, with the error bars included (±1σ). The left-hand axis is the average intermolecular H-bond counts (blue line), and minimum salt-bridge distances at the polar charged residues at two different sides of β-sheet in nm; Lys16-Glu22, Asp23 (green line), and Glu22, Asp23-Lys16 (red line).The horizontal axis is the separation of the centres of mass in nm.(modified from Mehrazma et al.)[1]

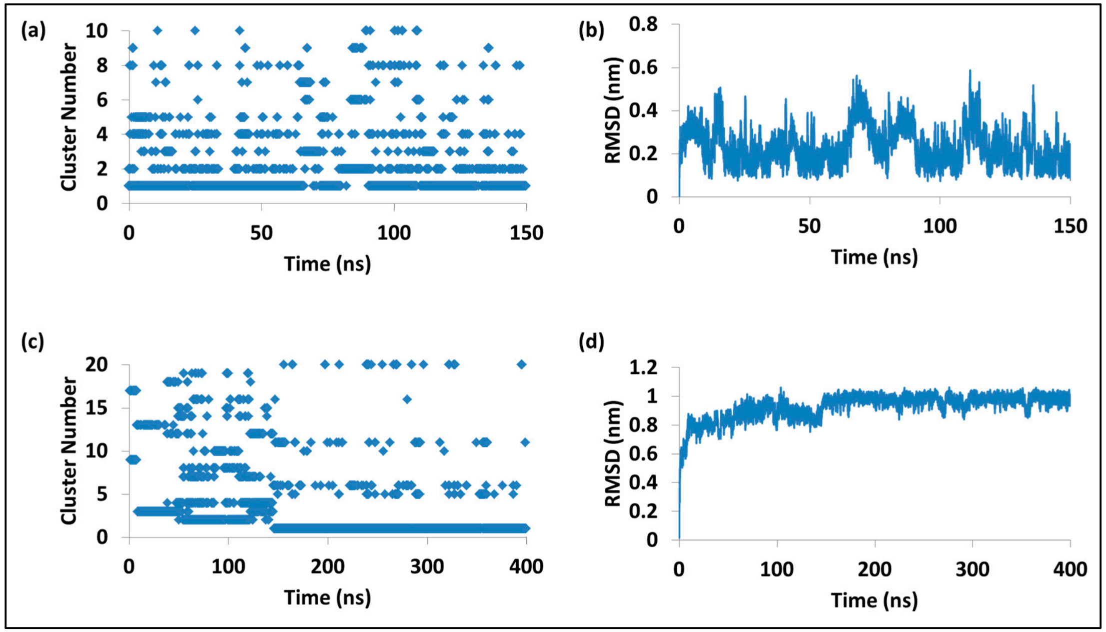

Figure S5 The backbone structure analysis for R^T-SGD1, (a) cluster analysis, (b) RMSD, and cluster analysis and also for R^B-SGD1 (c) cluster analysis (d) RMSD.

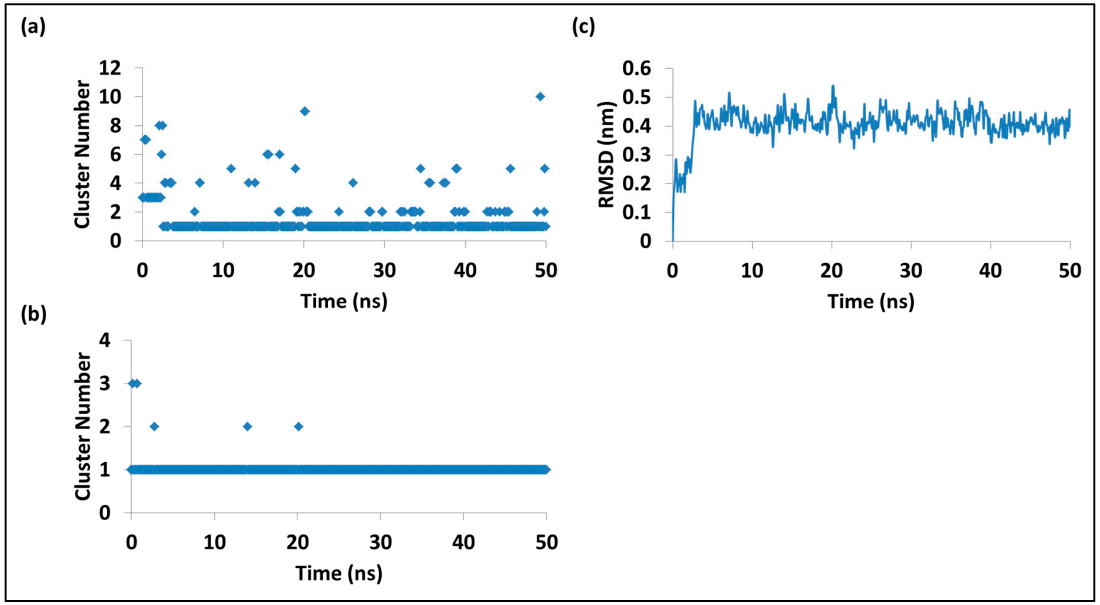

Figure S6 The structure analysis for SGB1 homodimer, (a) the clusters from all-atom based and (b) backbone based cluster analysis, (c) The RMSD calculation profile based on all-atom.

References:

(1) Mehrazma, B.; Petoyan, A.; Opare, S. K. A.; Rauk, A. Interaction of the N-AcAβ(13–23)NH2 Segment of the Beta Amyloid Peptide with Beta-Sheet-Blocking Peptides: Site and Edge Specificity. *Can. J. Chem.* **2016**, *6* (94), 583–592.

Table S2. Aβ_{42}-SGB1 energy analysis: Aβ-SGB1 (SGB1 = PP) energy analysis (kJ/mol). The clusters are listed by the hierarchy of their appearance in the trajectory.

Cluster number	P_i	V_{gas}(Aβ*)	V_{gas}(PP*)	V_{int}(PP*-Aβ*)	V_{gas}(PP-Aβ)	G_{PBSA}(PP-Aβ)	$G_{gas\text{-}PBSA}$(PP-Aβ)	$\Delta G_{gas\text{-}PBSA}$	$\Delta G_{LIE\text{-}D}$	$\Delta G_{LIE\text{-}DR}$
Ba1	0.80	4501 ±7	708 ±3	-349 ±5	4861 ±6	-2525 ±11	2335 ±13	78 ±29	-33 ±4	-26 ±41
Bb2	0.13	4793 ±17	611 ±3	-617 ±14	4787 ±16	-2439 ±138	2243 ±139	-14 ±33	-58 ±7	-102 ±43
Bb3	0.11	4754 ±5	615 ±2	-624 ±5	4745 ±5	-2544 ±20	2241 ±21	-16 ±141	-67 ±4	-106 ±41
Bb1	0.58	4706 ±8	629 ±1	-668 ±7	4666 ±7	-2445 ±16	2221 ±8	-37 ±32	-64 ±4	-101 ± 41
Bc1	0.12	4238 ±10	624 ±1	-402 ±1	4460 ±8	-2103 ±10	2357 ±12	100 ±29	-33 ±3	-37 ±41
Bc4	0.08	4490 ±5	576 ±1	-567 ±8	4499 ±7	-2192 ±35	2307 ±35	50 ±44	-60 ±5	-91 ±41
Monomer		V_{gas}(Aβ)	V_{gas}(PP)			G_{PBSA}	$G_{gas\text{-}PBSA}$			
Aβ		4376 ±11	-	-	-	-1915 ±24	2460 ±26	-		
SGB1		-	602 ±1	-		-805±1	-203 ±1	-		

Table S3. Aβ_{42}-SGD1 energy analysis: Aβ-SGD1 (SGD1 = PP) energy analysis: (kJ/mol). The clusters are listed by the hierarchy of their appearance in the trajectory.

Cluster number	P_i	V_{gas}(Aβ*)	V_{gas}(PP*)	V_{int}(PP*-Aβ*)	V_{gas}(PP-Aβ)	G_{PBSA}(PP-Aβ)	$G_{gas\text{-}PBSA}$(PP-Aβ)	$\Delta G_{gas\text{-}PBSA}$	$\Delta G_{LIE\text{-}D}$	$\Delta G_{LIE\text{-}DR}$
Da1	0.24	4459 ±5	706 ±1	-351 ±3	4814 ±4	-2476 ±13	2337 ±14	56 ±30	-37 ±3	-35 ±40
Da2	0.23	4499 ±15	707 ±1	-339 ±3	4866 ±11	-2539 ±22	2327 ±24	45 ±36	-35 ±3	-40 ±42
Db1	0.25	4584 ±23	617 ±4	-616 ±39	4586 ±32	-2288 ±12	2298 ±35	16 ±43	-48 ±4	-62 ±41
Db2	0.23	4584 ±7	616 ±1	-594 ±5	4606 ±6	-2313 ±56	2293 ±57	11 ±62	-47 ±5	-59 ±41
Dc1	0.62	4606 ±5	691 ±1	-663 ±5	4633 ±5	-1971 ±11	2656 ±12	374 ±29	-67 ±3	-111 ±40
Dd2	0.09	4651 ±42	597 ±1	-598 ±32	4650 ±6	-2334 ±15	2224 ±16	-58 ±31	-56 ±3	-69 ±41
Dd3	0.08	4589 ±19	593 ±2	-460 ±7	4722 ±14	-2481 ±13	2241 ±19	-41 ±32	-48 ±4	-62 ±44
Dd1	0.33	4476 ±11	611 ±1	-568 ±3	4519 ±8	-2180 ±17	2339 ±19	58 ±32	-54 ±3	-75 ±41
Dd5	0.03	4365 ±7	605 ±3	-419 ±3	4551 ±6	-2291 ±15	2261 ±16	-21 ±31	-41 ±3	-42 ±41
Monomer		V_{gas}(Aβ)	V_{gas}(PP)			G_{PBSA}	$G_{gas\text{-}PBSA}$			
Aβ		4376 ±11	-	-	-	-1915 ±24	2460 ±26	-		
SGD1		-	599 ±1	-		-778 ±1	-179 ±2	-		
